# Rapid *in vitro* activity of telavancin against *Bacillus anthracis* and *in vivo* protection against inhalation anthrax infection in the rabbit model

**DOI:** 10.1128/aac.00112-24

**Published:** 2024-06-18

**Authors:** William S. Lawrence, Jennifer E. Peel, Richard A. Slayden, Johnny W. Peterson, Wallace B. Baze, Martha E. Hensel, Elbert B. Whorton, David W. C. Beasley, Jason E. Cummings, Ines Macias-Perez

**Affiliations:** 1 Department of Microbiology and Immunology, University of Texas Medical Branch, Galveston, Texas, USA; 2 Department of Microbiology, Immunology & Pathology, Colorado State University, Fort Collins, Colorado, USA; 3 Department of Comparative Medicine and Research, University of Texas MD Anderson, Bastrop, Texas, USA; 4 Department of Epidemiology, University of Texas Medical Branch, Galveston, Texas, USA; 5 Product Development Division, Cumberland Pharmaceuticals, Nashville, Tennessee, USA; Tufts University-New England Medical Center, Boston, Massachusetts, USA

**Keywords:** inhalation anthrax, *Bacillus anthracis*, antibiotic, rabbit model, therapeutic

## Abstract

Inhalation anthrax is the most severe form of *Bacillus anthracis* infection, often progressing to fatal conditions if left untreated. While recommended antibiotics can effectively treat anthrax when promptly administered, strains engineered for antibiotic resistance could render these drugs ineffective. Telavancin, a semisynthetic lipoglycopeptide antibiotic, was evaluated in this study as a novel therapeutic against anthrax disease. Specifically, the aims were to (i) assess *in vitro* potency of telavancin against 17 *B. anthracis* isolates by minimum inhibitory concentration (MIC) testing and (ii) evaluate protective efficacy in rabbits infected with a lethal dose of aerosolized anthrax spores and treated with human-equivalent intravenous telavancin doses (30 mg/kg every 12 hours) for 5 days post-antigen detection versus a humanized dose of levofloxacin and vehicle control. Blood samples were collected at various times post-infection to assess the level of bacteremia and antibody production, and tissues were collected to determine bacterial load. The animals’ body temperatures were also recorded. Telavancin demonstrated potent bactericidal activity against all strains tested (MICs 0.06–0.125 μg/mL). Further, telavancin conveyed 100% survival in this model and cleared *B. anthracis* from the bloodstream and organ tissues more effectively than a humanized dose of levofloxacin. Collectively, the low MICs against all strains tested and rapid bactericidal *in vivo* activity demonstrate that telavancin has the potential to be an effective alternative for the treatment or prophylaxis of anthrax infection.

## INTRODUCTION

*Bacillus anthracis* is a gram-positive, spore-forming bacillus bacterium that causes anthrax infection. It is a Tier 1 select agent because of its infectivity at low doses, ease of mass production, potential for weaponization, and stability during dissemination (1). Depending on the route of exposure, anthrax infection manifests in four forms: cutaneous, gastrointestinal, inhalational, and injectional. Inhalation anthrax infection is the most detrimental form of the disease, with a fatality rate of up to 80% when either no treatment is given or treatment is delayed (2, 3). Initial symptoms of the disease are nonspecific and include fever, chills, body aches, fatigue, and nausea. Still, the symptoms worsen late in infection, and the infected individual develops dyspnea, pulmonary congestion, severe respiratory distress, hemoptysis, and ultimately shock (4, 5).

*B. anthracis* is of particular interest to the biodefense sector since it is one of the few biological agents that has been released or used on civilians as a nefarious act of bioterrorism (6–8). The World Health Organization has estimated that a *B. anthracis* spore release in a city of 5 million could cause up to 250,000 casualties with 100,000 deaths (9). Given this threat, the US Department of Health and Human Services is tasked with stockpiling medical countermeasures and ensuring “timely and accurate recommended utilization guidelines” to protect the public against anthrax (10). The ensuing mortality and morbidity would be further compounded with the use of *B. anthracis* strains that were genetically modified to be resistant to antibiotics approved by the Food and Drug Administration (FDA) currently used to treat anthrax infection, such as ciprofloxacin, doxycycline, and levofloxacin. Due to this potential threat, there remains a need for new antibiotic treatments to combat the disease. Such novel medical countermeasures (MCMs) should be developed and added to the Strategic National Stockpile in the event that current MCM stocks begin to demonstrate less or no activity as first-line antibiotic therapy for resistant strains developed for nefarious purposes.

Telavancin (TD-6424, trade name Vibativ) is the only clinically approved semisynthetic glycopeptide antibiotic derived from vancomycin. It differs significantly from its parent structure by the decylaminoethyl modification on the vancosamine unit, a modification that is responsible for telavancin’s enhanced potency against gram-positive strains (11, 12).

Telavancin has a dual mode of action. First, it retains the mechanism of action of vancomycin by binding lipid II, thereby inhibiting bacterial cell wall biosynthesis (13, 14). This interaction is promoted by the decylaminoethyl lipid, which anchors into the cytoplasmic membrane and brings telavancin into proximity with peptidoglycan precursors. For this reason, telavancin displays a higher binding affinity for the bacterial cell surface and increased inhibition of transglycosylation (14). Telavancin’s lipid moiety is responsible for the second mode of action: the concentration-dependent dissipation of bacterial cell membrane potential (at 10-fold MIC), leading to membrane permeabilization and leakage of ATP and potassium ions (13–15). Telavancin displays a low propensity to induce spontaneous resistance in staphylococci and enterococci (16). Interestingly, this dual mechanism of action gives telavancin a potency 10-fold greater than vancomycin (17–19).

In the US and Canada, telavancin was approved in September 2009 for use in the treatment of complicated skin and skin structure infections (CSSSIs) caused by susceptible gram-positive species such as *Staphylococcus aureus*, *Streptococcus agalactiae*, *Streptococcus pyogenes*, and *Enterococcus faecalis* and in June 2013 for use in the case of hospital-acquired pneumonia including ventilator-associated pneumonia caused by *S. aureus* (20). Telavancin is active against a variety of gram-positive species, including methicillin-resistant *Staphylococcus aureus* (MRSA) (MIC = 0.016–0.125 mg/L), VanB-type vancomycin-resistant enterococci (VRE) (MIC = 2 mg/L), and *Streptococcus pneumoniae* (MIC = 0.008–0.03 mg/L) (17, 21–23). Many species of streptococci (including multidrug-resistant and penicillin-resistant strains) and *Listeria* (MIC, 0.125 mg/L) are susceptible to telavancin (21, 24–26). Unlike teicoplanin, telavancin is also potent against vancomycin-intermediate *Staphylococcus aureus* (VISA) strains (17, 27). Telavancin is known to exert activity against *S. aureus* harbored intracellularly inside murine THP-1 and human J774 macrophage cell lines (28). Biofilm-generating *S. aureus* and *Staphylococcus epidermidis* are also susceptible to the antibacterial effects of telavancin (29). *Actinomyces* species (MIC, 0.125–0.25 mg/L), *Clostridium difficile* (MIC, 0.125–0.5 mg/L), and many other anaerobic bacteria are susceptible to telavancin (30).

Telavancin was tested against *B. anthracis* in two separate *in vitro* experiments. The first tested 15 strains (31), and the current study tested 17 strains. Both demonstrate that telavancin has potent activity against all *B. anthracis* strains with MIC at or below 0.125 mg/L.

These results led us to test the protective efficacy of telavancin against inhalation anthrax infection using the rabbit model. The *in vivo* study was performed using the rabbit model, which is a superior model for inhalation anthrax infection relative to rodent models (32, 33) and was conducted as a trigger-to-treat study; therefore, treatment began after *B. anthracis* protective antigen (PA) was detected in each animal’s serum via electrochemiluminescence. Blood and tissue samples were also collected to assess the extent of infection and the effectiveness of telavancin. To our knowledge, this is the first reported work showing the therapeutic potential of telavancin against inhalation anthrax in a large animal model.

## RESULTS

### Telavancin *in vitro* activity against *B. anthracis* strains

Telavancin was screened against a diverse panel of *B. anthracis* strains consisting of laboratory and clinical strains considered representative of the drug susceptibility spectrum associated with clinical infections (34). Telavancin has a minimal inhibitory concentration range of 0.0625–0.125 mg/L against the laboratory reference strain and the panel of clinical strains with various susceptibilities to standard-of-care drugs (Table 1). This MIC value and inhibition are characteristic of clinical drugs with activity against *B. anthracis*, which is within the range for a drug to have efficacy *in vivo* (35). Most significantly, telavancin demonstrated superior potency to current clinically used drugs to treat *B. anthracis* infections with no observable *in vitro* spontaneous drug resistance in MIC assays (36).

**TABLE 1:** MIC summary for telavancin and doxycycline against *B. anthracis* strains^*a*^

Strain	Telavancin	Doxycycline
Ames 3838	<0.0625	0.03125
Graves	<0.0625	0.03125
46-PY-5	<0.0625	0.03125
Ames	<0.0625	0.03125
Kruger B (A0442)	0.125	0.03125
Vollum (A0488)	<0.0625	<0.0156
WNA	<0.0625	0.03125
A0318	<0.0625	0.03125
A0471	<0.0625	0.03125
Anthrax strain collection (ASC) 506	<0.0625	0.03125
ASC 525	<0.0625	0.03125
2000032823 (CDC #1)	<0.0625	<0.0156
2002734753 (CDC #2)	0.125	0.03125
2010719149 (CDC #3)	<0.0625	0.03125
2006200760 (CDC #4)	<0.0625	<0.0156
ASC 32	<0.0625	0.03125
ASC 149	<0.0625	0.03125

^*a*^ Data represented as mg/L.

### Infection and toxemia

The mean aerosol exposure dose of *B. anthracis* spores for 30 animals was 2.25 × 10^7^ cfu (±4.03 × 10^5^), corresponding to 225 LD_50_ (50% lethal dose), and the mean duration of the aerosol challenges was 13 min. Analysis of sera extracted from whole blood samples collected every 6 hours beginning 12 hours post-infection showed that exposed animals exhibited toxemia [as measured by detectable levels of PA using an electrochemiluminescence (ECL) assay] as early as 18 hours post-infection and as late as 30 hours post-infection (7%). However, the majority (63%) of the animals were positive for PA at 18 hours post-infection (Table 2). The concentration of PA in the sera ranged from approximately 50 to 2,000 picograms per milliliter (pg/mL).

**TABLE 2:** Time points of PA detection

Treatment group	Animal ID#	Time of PA detection	PA concentration (pg/mL)
Telavancin	8629	18	960
	8630	18	597
	8633	18	824
	8637	18	371
	8639	18	796
	8641	24	348
	8643	18	1,781
	8646	24	2,039
	8647	24	639
	8651	18	418
	8653	24	841
	8656	18	377
Levofloxacin	8628	18	1,052
	8632	18	1,128
	8634	18	58.4
	8635	24	468
	8638	18	146
	8640	18	154
	8642	24	514
	8644	30	258
	8649	24	1,189
	8652	18	1,229
	8654	18	146
	8657	24	269
Saline	8631	18	509
	8636	30	1,007
	8645	18	922
	8648	18	389
	8650	18	746
	8655	24	164

### Survival

**Fig 1:**
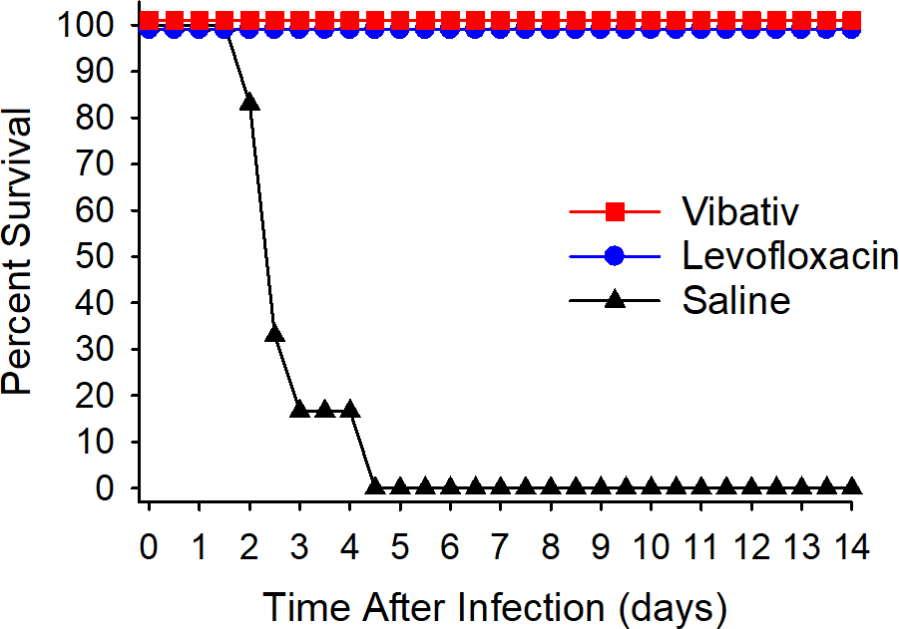
The therapeutic efficacy of telavancin and levofloxacin in the rabbit model of inhalation anthrax. New Zealand White rabbits were challenged with 225 LD_50_ *B. anthracis* Ames spores via inhalation. Telavancin treatment was initiated upon the detection of PA in the animals’ sera and was administered at 30 mg/kg twice daily for 5 days. Levofloxacin, at 12.5 mg/kg, administered once daily for 5 days, and saline, administered once daily, were used as controls. Survival was monitored for 14 days post-infection. The percent survival rates for the two antibiotic-treated groups were significantly (*P* < 0.001) higher than that of the saline-treated group.

Following infection, the animals were monitored at least twice daily for 14 days. The most common clinical signs included anorexia, lethargy, and respiratory distress. These observations were consistent with symptoms of inhalational anthrax. The telavancin-treated group exhibited 100% survival after the challenge, which was significantly (*P* ˂ 0.001) greater than that of the saline-treated group, which showed no survival (Fig. 1; Table S1). As expected, the animals administered saline succumbed to infection 2–4 days post-infection. The group treated with the humanized levofloxacin dose (12.5 mg/kg) also showed 100% survival. These results indicate that telavancin was completely protective in this model of inhalation anthrax infection, and it was noninferior to the regimen of levofloxacin that was used as a positive control in this study.

### Temperature response during infection and treatment

The antibiotic-treated groups (telavancin and levofloxacin) exhibited comparable temperature responses after the challenge (Fig. S1**;** Table S2). Specifically, animals in both groups had febrile responses at approximately Day 1 post-infection that peaked at nearly 41°C before Day 2. By Day 2, the mean temperatures for the antibiotic-treated groups returned to baseline, most likely due to treatment, which began at 18–30 hours post-infection. Interestingly, both antibiotic-treated groups appeared to have minor secondary febrile responses from 7 to 9 days post-infection, which was after treatment was ended (Day 6), but these temperature elevations subsided by Days 10–11. The mean temperature of the animals treated with saline also began to rise by Day 1, and it remained elevated until hours before the animals succumbed to infection. These results show that post-exposure treatment with telavancin was effective at reducing and resolving the febrile response consistently reported with anthrax infection.

### Bacteremia

**Fig 2:**
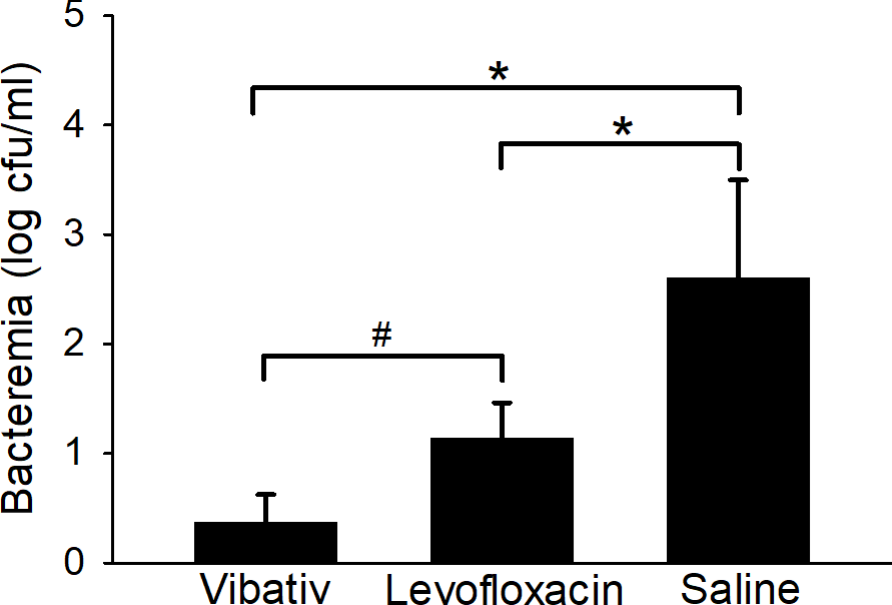
*B. anthracis* bacteremia levels during infection and antibiotic treatment at 24 hours post-infection. New Zealand White rabbits were challenged with 225 LD_50_ *B. anthracis* Ames spores via inhalation. Telavancin treatment was initiated upon the detection of PA in the animals’ sera and was administered at 30 mg/kg twice daily for 5 days. Levofloxacin, at 12.5 mg/kg, was administered once daily for 5 days, and saline, administered once daily, was used as a control. Whole blood was plated on TSAII plates and incubated at 37°C for 16–24 hours. Data are presented as averages with standard error bars. The asterisk and hashtag indicate *P* < 0.05 and *P* < 0.10, respectively, differences between groups.

Table 3 indicates the *B. anthracis* bacterial load in the blood of each animal in the three treatment groups. The data are presented in colony-forming units per milliliter (cfu/mL). Bacteremia was detected as early as 24 hours post-infection. At that time, the average levels of bacteremia for the antibiotic-treated (telavancin and levofloxacin) groups were significantly (*P* ˂ 0.05) lower than that of the saline-treated group (Fig. 2; Table S3). Approximately half of the animals in both the telavancin- and levofloxacin-treated groups received their initial treatment by 24 hours post-infection. Interestingly, only 2 out of 12 animals treated with telavancin were positive at this time point, but 8 out of 12 animals treated with levofloxacin were positive at this same time. Moreover, when comparing only the two antibiotic-treated groups, the average level of bacteremia was slightly lower (*P* < 0.10) among the animals treated with telavancin (Fig. 2; Table S3), which is suggestive that the humanized telavancin dosage was more effective at eliminating the bacteria in the blood relative to the humanized levofloxacin dosage used. By 48 hours post-infection, all antibiotic-treated animals were negative for bacteremia and remained so for the remainder of the post-infection period (Days 7, 10, and 14 post-infection, not shown). Overall, these results suggest that rabbits treated with telavancin more rapidly clear *B. anthracis* from circulation than rabbits treated with levofloxacin.

**TABLE 3:** *B. anthracis* bacteremia levels^*a*^

Group	Animal ID#	−7 days	12 hrs PI	24 hrs PI	48 hrs PI	72 hrs PI	96 hrs PI	120 hrs PI
Telavancin	8629	0	0	0	0	0	0	0
	8630	0	0	0	0	0	0	0
	8633	0	0	0	0	0	0	0
	8637	0	0	0	0	0	0	0
	8639	0	0	0	0	0	0	0
	8641	0	0	0	0	0	0	0
	8643	0	0	0	0	0	0	0
	8646	0	0	3.90*E* + 02	0	0	0	0
	8647	0	0	0	0	0	0	0
	8651	0	0	0	0	0	0	0
	8653	0	0	7.00*E* + 01	0	0	0	0
	8656	0	0	0	0	0	0	0
Levofloxacin	8628	0	0	1.00*E* + 01	0	0	0	0
	8632	0	0	7.00*E* + 01	0	0	0	0
	8634	0	0	0	0	0	0	0
	8635	0	0	1.00*E* + 01	0	0	0	0
	8638	0	0	0	0	0	0	0
	8640	0	0	0	0	0	0	0
	8642	0	0	2.00*E* + 01	0	0	0	0
	8644	0	0	0	0	0	0	0
	8649	0	0	4.42*E* + 03	0	0	0	0
	8652	0	0	3.60*E* + 02	0	0	0	0
	8654	0	0	2.00*E* + 01	0	0	0	0
	8657	0	0	1.00*E* + 01	0	0	0	0
Saline	8631	0	0	1.90*E* + 05	4.20*E* + 04	Expired		
	8636	0	0	0	9.55*E* + 02	8.50*E* + 03	1.76*E* + 06	Expired
	8645	0	0	6.71*E* + 03	4.29*E* + 04	5.60*E* + 06	Expired	
	8648	0	0	2.50*E* + 02	8.20*E* + 05	Expired		
	8650	0	0	1.16*E* + 04	1.45*E* + 06	Expired		
	8655	0	0	0	1.10*E* + 06	Expired		

^*a*^ Data represented as cfu/mL; PI, post-infection.

### Bacterial load in tissues

**Fig 3:**
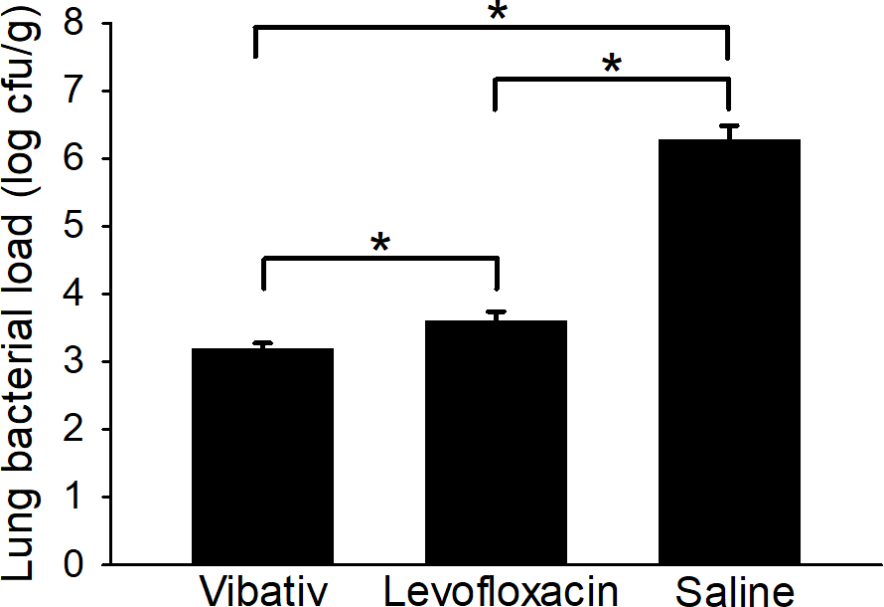
*B. anthracis* bacterial load in lung tissue at time of euthanasia. New Zealand White rabbits were challenged with 225 LD_50_ *B. anthracis* Ames spores via the inhalation route. Telavancin treatment was initiated upon the detection of PA in the animals’ sera and was administered at 30 mg/kg twice daily for 5 days. Levofloxacin, at 12.5 mg/kg, and 0.9% sodium chloride solution were each administered once daily for 5 days and served as the positive and negative controls, respectively. Tissues were homogenized, and the homogenates were plated on TSAII plates. The plates were then incubated at 37°C for 16–24 hours. Data are presented as averages with standard error bars. The asterisk indicates a significant (*P* < 0.05) difference between groups.

Table S4 gives the *B. anthracis* bacterial load in the lung tissue collected from each animal in the three treatment groups at the time of euthanasia due to reaching humane or scientific endpoints. Although *B. anthracis* was also detected in the brains, mediastinal lymph nodes, and spleens of the saline-treated control animals (average bacterial loads of 6.68 × 10^5^ cfu/g, 2.59 × 10^6^ cfu/g, and 6.43 × 10^6^ cfu/g, respectively), no bacteria were recovered from these tissues from any animal in either group treated with telavancin or levofloxacin. In a comparison of *B. anthracis* bacterial loads in lung tissue, there were significantly (*P* < 0.05) fewer bacteria in the two antibiotic-treated groups relative to the saline control group (Fig. 3; Table S4). When comparing the two antibiotic-treated groups, the average bacterial load in the lung tissue of the animals treated with telavancin (1.93 × 10^3^ cfu/g or 3.19 logs) was significantly (*P* ˂ 0.05) lower than that of the animals treated with levofloxacin (6.93 × 10^3^ cfu/g or 3.60 logs) (Fig. 3; Table S4), suggesting once again that the humanized telavancin dosage was more effective at eliminating the bacteria in tissues relative to the humanized levofloxacin dosage. Since tissues were not collected for the assessment of bacterial load at various scheduled times post-infection, it is unknown whether telavancin completely prevented the migration of *B. anthracis* to the tissues or if it cleared the bacteria after the bacteria entered the tissues. Nonetheless, these results show that telavancin was more effective compared to levofloxacin in preventing, clearing, and reducing *B. anthracis* infection of tissues after exposure to inhalation anthrax.

### Assessment of anti-PA antibody levels

**Fig 4:**
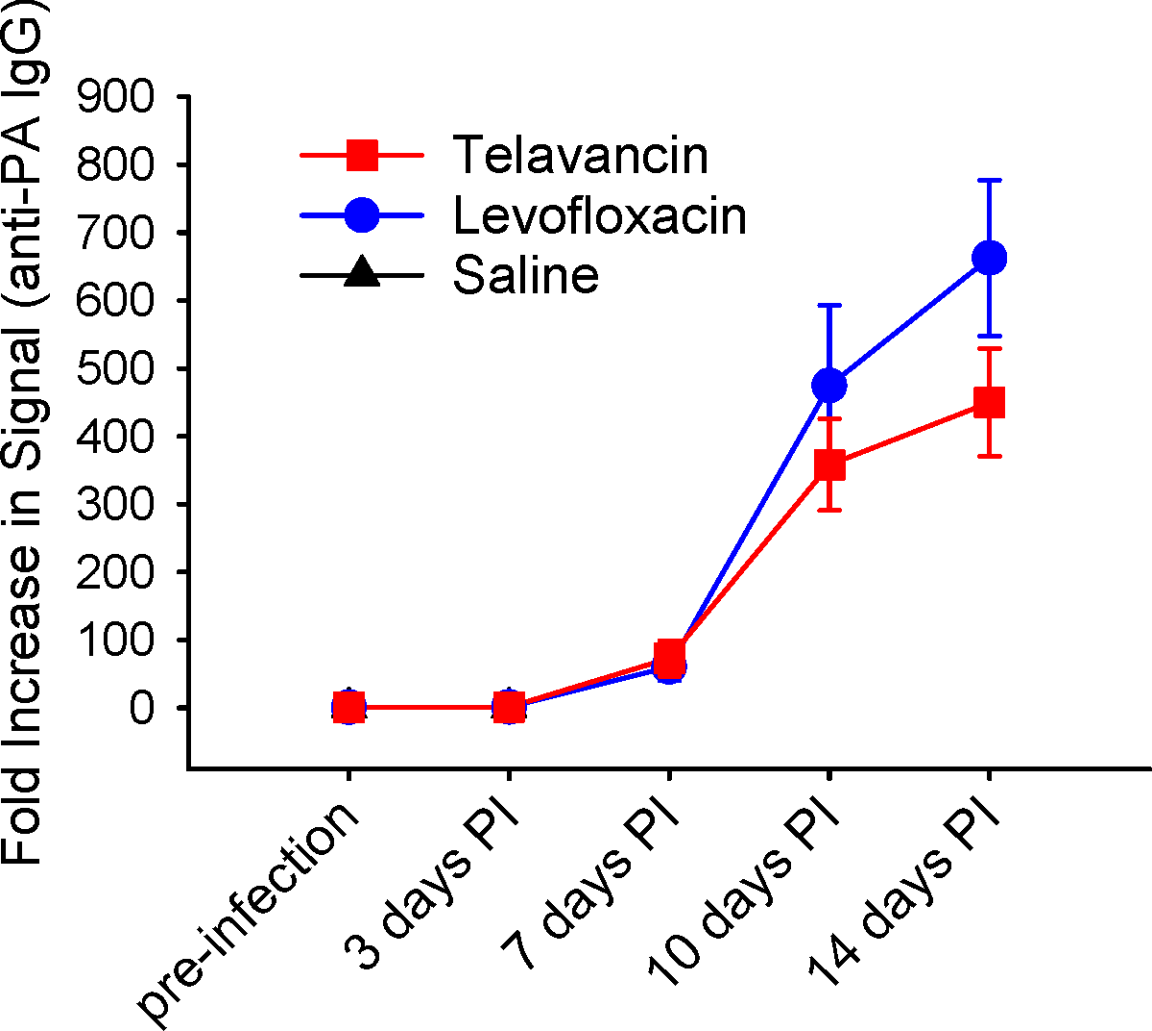
Anti-PA antibody response during infection and antibiotic treatment. New Zealand White rabbits were challenged with 225 LD_50_ *B. anthracis* Ames spores via inhalation. Telavancin treatment was initiated upon the detection of PA in the animals’ sera and was administered at 30 mg/kg twice daily for 5 days. Levofloxacin, at 12.5 mg/kg, was administered once daily for 5 days, and saline, administered once daily, was used as control. Sera were extracted from whole blood at various time points, and the anti-PA antibody response was measured in the sera via ECL. Data are presented as averages with standard error bars.

The animals in the two antibiotic-treated groups began to develop antibody responses to *B. anthracis* protective antigen as early as 7 days post-infection, and the responses intensified at Days 10 and 14 post-infection (Fig. 4; Table S5). There was no significant difference in average serum anti-PA IgG titers between the telavancin- and levofloxacin-treated groups; however, both antibiotic-treated groups had a significant increase (*P* < 0.05) over time. These results demonstrate that telavancin did not alter the humoral immune response to PA generated following antibiotic treatment of anthrax infection.

### Necropsy and histopathology

At necropsy, the animals in the saline-treated group consistently showed blackened, enlarged, and hemorrhagic mediastinal lymph nodes, discoloration, and diffuse hemorrhage of the lungs with pleural effusion. There was also pericardial effusion and multifocal hemorrhage on the serosal surface of the vermiform appendix. Additional notable findings, although less consistent, in the saline-treated group were the presence of hemorrhage in the meninges, multifocal discoloration of the cecum, and hemorrhage in the thymus. Importantly, all these findings have been reported to be associated with anthrax infection in animal models (37–42). The gross pathology observed among the groups treated with telavancin and levofloxacin was far less prominent than in the untreated control animals. Animals in these antibiotic-treated groups still exhibited a few pathologic findings, such as pulmonary congestion, multifocal hemorrhage of the lungs, and enlarged mediastinal lymph nodes, indicating an active infection, but the lesser pathology among these groups relative to the untreated controls serves as evidence of disease mitigation.

**Fig 5:**
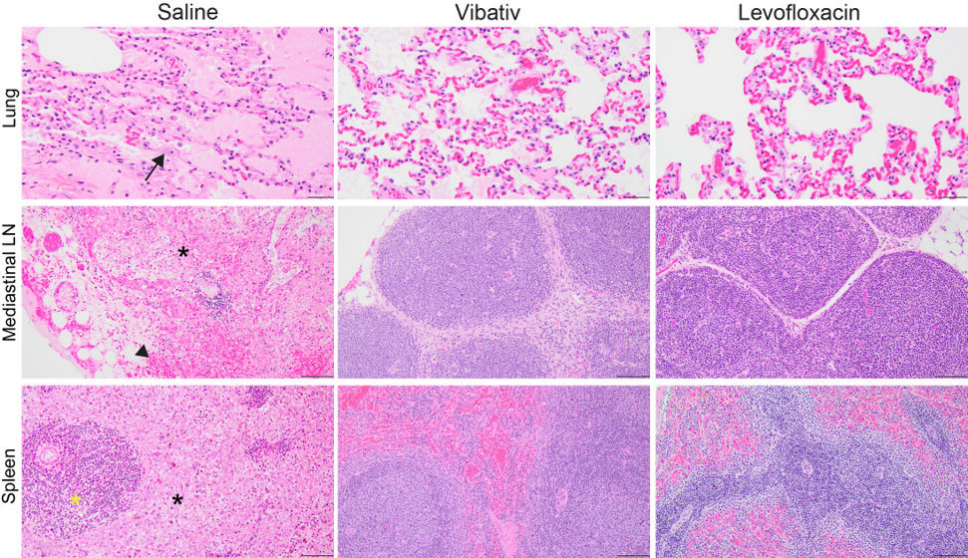
Histopathological lesions in lungs, spleen, and lymph nodes of rabbits infected with inhalation anthrax and treated with telavancin and levofloxacin. New Zealand White rabbits were inoculated with anthrax via the inhalation route. Lung, mediastinal lymph node, and spleen were collected for routine histopathology. Significant lesions were only detected in the animals dosed with saline. Lung: The alveoli contain edema fluid. Bacteria are within the alveoli vasculature (arrow). Inset: Higher magnification of intravascular anthrax bacilli. Mediastinal lymph node: Diffusely, the lymphoid follicles are necrotic (*) with areas of hemorrhage (arrowhead). Spleen: The white pulp (arrowhead) and red pulp (black asterisk) are diffusely necrotic. Hematoxylin and eosin. Scale bar = 20 µm.

Histopathological analysis showed notable lesions in the tissues of the control animals, with the lesions in the lungs, mediastinal lymph nodes, and spleen being the most significant (Fig. 5). The remaining collected tissues in the control animals also exhibited pathology, albeit to a lesser extent. Specifically, there was evidence of degeneration and necrosis in the heart and liver; bacteria were present in the liver, kidneys, and brain, and hemorrhage was present in the brain (Fig. S2). Conversely, tissues from animals treated with telavancin and levofloxacin showed only evidence of disease mitigation, and there were no substantial differences among the tissues from these two groups (Fig. 5; Fig. S2). These results show that telavancin treatment effectively clears *B. anthracis* from tissues, ultimately leading to disease mitigation and survival.

## DISCUSSION

Telavancin (TD-6424) is a lipoglycopeptide derivative of vancomycin administered once daily as a 1-hour infusion at a dose of 10 mg/kg/day for the treatment of CSSSIs and nosocomial pneumonia (hospital-acquired and ventilator-associated) in adults with a CrCl > 50 mL/min. Telavancin has excellent bactericidal activity against aerobic and anaerobic gram-positive bacteria, including multiple resistant phenotypes of *S. aureus,* and superior efficacy compared to vancomycin in diverse animal models of difficult-to-treat gram-positive infections, such as pneumonia, bacteremia, and endocarditis (17, 20, 43–45). Although the bactericidal activity of telavancin against many clinically relevant gram-positive bacteria has been demonstrated (27, 46), limited data are available on *B. anthracis*. Here, we investigated the *in vitro* activities of telavancin against 17 strains of *B. anthracis*. In our study, telavancin exhibited potent bactericidal activity against the 17 *B. anthracis* strains tested with MICs < 0.0625 mg/L for all but two strains with MIC of 0.125 [Kruger B (A0442) and 2002734753 (CDC #2)] similar to the FDA-approved and comparator agent, doxycycline and levofloxacin, parenteral procaine penicillin G, and ciprofloxacin. Notably, the susceptibility profile of these diverse strains demonstrated resistance and intermediate susceptibility across multiple families of antibiotics and combinations (34), underscoring the importance of expanding the spectrum of antibiotics available for the treatment of anthrax.

These encouraging *in vitro* results led us to assess the protective efficacy of telavancin *in vivo*. In the present study, telavancin was shown to be completely protected against inhalation anthrax infection using New Zealand White rabbits challenged with 225 LD_50_ *B. anthracis* Ames spores. Moreover, this study was conducted as a trigger-to-treat study wherein telavancin treatment was not initiated until the infection was confirmed by the detection of the *B. anthracis* PA. This aspect would be beneficial for real-time application if a biomarker such as PA was used to definitively diagnose anthrax infection in the early stages of infection. Future studies aimed at determining the length of time telavancin treatment could be delayed after infection and still provide protection would be useful since this would further mimic real-world cases wherein patients seek treatment in the late stages of infection. However, assessing the level of protection afforded by telavancin at various times post-infection was beyond the scope of the current study.

Results showed that telavancin could quickly eliminate *B. anthracis* from circulation, as evidenced by the absence of bacteremia and the waning fever response 1 day after the start of treatment. Interestingly, the level of bacteremia at 24 hours post-infection was slightly lower for the telavancin-treated group compared to our positive control, the levofloxacin-treated group, which is suggestive that telavancin was more effective than levofloxacin in eliminating the bacteria. Telavancin was also able to clear the bacteria more effectively from select tissues compared to levofloxacin, although tissue clearance may partly be due to an animal’s own acquired immunity since tissues of the surviving animals were harvested 14 days post-infection. A robust anti-PA IgG antibody response was evident 7–10 days post-infection. *B. anthracis* was, for instance, detected in the lungs of both antibiotic-treated animals at termination. However, the bacterial load was significantly less than that of the saline-treated animals. Interestingly, the telavancin-treated group had a lower bacterial load in the lungs compared to the levofloxacin group, which would suggest once again that telavancin was more effective than levofloxacin at eliminating the bacteria. The *B. anthracis* spores were hypothesized to persist in the lungs for the entire post-infection period, but the prolonged presence of spores in the lungs after an initial exposure is a characteristic feature of inhalation anthrax. In fact, dormant *B. anthracis* spores have been recovered from the lungs of nonhuman primates (47) and mice (48, 49) weeks or months post-exposure. This is a possible explanation for this study’s mild secondary fever responses observed among the antibiotic-treated groups. Based on these observations, the CDC recommends administering post-exposure prophylaxis antimicrobial therapy for 60 days (50).

The CDC recently sponsored and published an Anthrax Preparedness supplement (51) with over a dozen articles in response to bioterrorism concerns, given the potential for *B. anthracis* to be weaponized. Since prospective studies in humans are limited, the CDC evaluated the effectiveness of various classes and combinations of antimicrobials by reviewing the outcomes of over 600 patients hospitalized with anthrax published over the last century (52) and concluded combination therapy to be superior to monotherapy for inhalation anthrax; however, neither monotherapy nor combination treatment is effective against anthrax meningitis. These findings highlight the unmet need for anthrax meningitis and *in vivo* studies to evaluate new antimicrobials and determine whether these new and current antianthrax agents penetrate the blood–brain barrier to be effective treatments for anthrax meningitis. The efficacy of telavancin (30 mg/kg) was evaluated in a rabbit model of meningitis caused by a strain of penicillin-resistant pneumococcus and compared with a combination of ceftriaxone (100 mg/kg) and vancomycin (20 mg/kg). Telavancin produced a more rapid lowering of cerebrospinal fluid (CSF) titers (−0.84 CFU/mL·h) than co-administration of vancomycin and ceftriaxone (−0.61 CFU/mL·h) (53), and the CSF was sterilized in 6 out of 10 rabbits by telavancin and only 4 out of 10 rabbits in the comparator arm. Of note, it was reported that the bacterial load at the initiation of therapy was significantly higher in the telavancin arm. These results warrant further studies with telavancin in a similar model of anthrax meningitis. Currently, there is a need for more treatment studies for anthrax meningitis in animal models (54). Future anthrax meningitis studies with telavancin in primates would be the most applicable to humans.

The CDC’s retrospective outcome study also underscores efforts to evaluate new combination therapies for synergy will be critical for successful anthrax preparedness. The combination of telavancin administered along with piperacillin-tazobactam, cefepime, imipenem, or ciprofloxacin for infections caused by single isolates of MRSA, VISA, vancomycin-resistant *Staphylococcus aureus* (VRSA), *S. agalactiae*, vancomycin-susceptible *E. faecalis*, vancomycin-resistant *Enterococcus faecium*, and daptomycin-resistant *S. aureus* reported no antagonism. When combinations of telavancin + piperacillin-tazobactam or telavancin + imipenem were employed against isolates of VISA, synergistic activity was reported. A synergistic effect was also recorded when telavancin was administered along with piperacillin-tazobactam, cefepime, or imipenem against a VRSA isolate (55). These findings strongly indicate a need to progress well-designed animal model treatment studies with telavancin to determine which combination with other antianthrax agents results in improved survival.

In conclusion, telavancin has potent *in vitro* activity against *B. anthracis* and is protective against lethal inhalation anthrax infection in the rabbit model. This widely used model mimics the human sequela associated with anthrax infection. Moreover, telavancin rapidly prevented disease progression and dissemination of the bacteria to the bloodstream and tissues. With its long half-life in humans, a dual mechanism of action, which differs from currently approved anti-anthrax agents, and synergistic activity, telavancin would be an ideal candidate for use as a medical countermeasure to inhalation anthrax infection. The results obtained with telavancin support further study to determine the protective dose in the nonhuman primate model of *B. anthracis* infection.

## MATERIALS AND METHODS

### *B. anthracis* strains

The 17 *B. anthracis* strains used in this study are those specified in the defined panels of Category A pathogens used for drug candidate evaluation in the NIAID drug screening (56) and included the Ames reference strain (NR3838) and clinically derived strains (34, 56).

### Telavancin *in vitro* activity to *B. anthracis* strains

MICs were determined in triplicate by the broth microdilution method in cation-adjusted Mueller–Hinton broth according to the methodology of the Clinical and Laboratory Standards Institute (57, 58). Vibativ was dissolved in a formulation supplied by Cumberland Pharmaceuticals (Nashville, TN) at a concentration of 6.4 mg/mL and diluted to 256 mg/L in the first column of a 96-well plate (final concentration was 128 mg/L when diluted 1:2 with culture). After 16–20-hour incubation at 37°C, the MICs were determined visually. The quality control strain *Escherichia coli* ATCC 25922 was tested in parallel for 16–18 hours.

### Animals

New Zealand White rabbits (Envigo, Denver, PA), half males and half females, weighing approximately 3.0–3.5 kg, were surgically implanted with venous access ports (VAPs) by the vendor to facilitate the collection of blood samples and the intravenous administration of the treatments. Upon receipt, the animals were surgically implanted with DST micro-T temperature data loggers (Star-Oddi Ltd, Gardabaer, Iceland) to record the animals’ temperature during the study. The animals were housed in ventilated cages with *ad libitum* access to food and water, and the room was maintained on a 12-hour light–dark cycle.

### Bacterial spores

*B. anthracis* Ames (NR3838) spores were grown in modified Schaeffer’s medium using a New Brunswick fermenter. After inoculation, the fermenter was operated with aeration, and the pH was maintained at pH 7.0–7.5 for approximately 4 days, after which the crude spores were harvested aseptically by centrifugation. The spores were then washed with sterile molecular-grade water. The spores were purified by density gradient centrifugation using sterile MD-76, a radiopaque contrast agent that can be used for density gradient centrifugation in spore purification. Visual observation of the spores at 400× by phase-contrast microscopy during each purification step was performed to ensure the production of a homogeneous suspension of highly refractile spores.

### Bacterial infection

New Zealand White rabbits were anesthetized with ketamine/xylazine and challenged by aerosol with 225 LD_50_ of *B. anthracis* Ames spores (2.25 × 10^7^ CFU) using a Biaera aerosol control platform (Biaera Technologies, Hagerstown, MD) fitted with a head-only aerosol exposure chamber. Real-time plethysmography (Data Sciences International, St. Paul, MN) was performed on each animal during the aerosol exposure to monitor respiration. A six-jet Collison nebulizer (CH Technologies, Westwood, NJ) was used to generate the aerosol. Aerosol samples were collected during each aerosol run using aerosol BioSamplers (SKC, Eighty-Four, PA) to confirm the challenge dose of spores for each animal by serial dilution and plating onto trypticase soy II agar plates containing 5% sterile sheep blood (TSAII). The duration of the aerosol delivery was based on the animal’s respiration and the total volume of inspired air. During the post-infection period, any animal with labored breathing, severe lethargy, or immobility was humanely euthanized according to AVMA guidelines. The experimental endpoint of the study was 14 days post-infection.

### Antibiotic treatment

Telavancin (Cumberland Pharmaceuticals, Nashville, TN), provided as a lyophilized powder, was reconstituted in sterile, pyrogen-free water to a concentration of 25 mg/mL and administered intravenously to the animals at a dose concentration of 30 mg/kg twice daily for 5 days, which mimics the levels measured in the serum of humans (59). Telavancin treatments were prepared fresh daily. Levofloxacin (Akorn Pharmaceuticals, Lake Forest, IL) at a concentration of 25 mg/mL was administered intravenously to the animals at a clinically equivalent dose of 12.5 mg/kg once daily for 5 days. The untreated control animals were given saline once daily for 5 days. Treatments were initiated upon detecting PA in the animals’ sera (point of antigenemia).

### Detection of PA

Using a rapid PA-ECL screening assay (MesoScale Discovery, Gaithersburg MD), the presence of PA was monitored in the animals’ sera that were extracted from whole blood (via the VAP) collected at various time points before and after infection. The time when PA was first detected was considered the point of antigenemia (PA in the blood) that dictated treatment initiation. A standard curve (0–100 ng/mL; *R*-squared value of 0.99) was analyzed in parallel for each assay to extrapolate the PA concentration of the serum samples. The limit of detection was 0.04 ng/mL. Test samples were assayed in duplicate.

### Assessment of bacteremia and bacterial load

The concentration of *B. anthracis* was measured in whole blood samples collected via the VAP at various time points before and after infection. Whole blood was plated onto TSAII plates using an automatic serial diluter and plater (Interscience Laboratories, Woburn, MA). After incubating the plates at 37°C for 16–24 hours, bacterial colonies were enumerated using an automatic colony counter (Interscience Laboratories, Woburn, MA). Bacterial colonies having morphology typical of *B. anthracis* were subcultured and confirmed as *B. anthracis* with bacteriophage ɣ.

Bacterial load was determined in each animal’s lung, lymph node (mediastinal), brain, and spleen. These tissues were homogenized in sterile water using a Stomacher 80 MicroBiomaster (Seward Ltd, Bohemia, NY). The homogenates were serially diluted in water and plated onto TSAII plates using an automatic diluter/plater and incubated at 37°C for 16–24 hours. Bacterial colonies were enumerated using an automated colony counter (Interscience Laboratories, Woburn, MA). Bacterial colonies having morphology typical of *B. anthracis* were subcultured and confirmed as *B. anthracis* with bacteriophage ɣ. The reported bacterial loads in tissues may be a combination of both vegetative bacteria and spores since the actual spore load was not determined.

### Assessment of anti-PA antibody response

Anti-PA IgG was measured in serum via ECL, similar to the PA-ECL screening assay. Biotinylated recombinant PA83 (List Biological Labs, Campbell, CA) was bound to streptavidin-coated plates (MesoScale Discovery, Gaithersburg, MD) and used as the capture antigen. Detection was accomplished using Sulfo-tag labeled anti-rabbit antibody and read buffer (MesoScale Discovery, Gaithersburg, MD). Results were presented as a fold increase in signal relative to the pre-challenge serum samples.

### Necropsy and histopathology

Gross pathology was performed on all animals, and tissues (lung, mediastinal lymph nodes, brain, and spleen) were collected and perfused with 10% phosphate-buffered formalin. Tissue sections were processed, embedded in paraffin, sectioned, stained with hematoxylin and eosin, and evaluated by microscopy. For the analysis of the stained tissue sections, a four-level severity scale was used when applicable utilizing the following terms: minimal (1 of 4), mild (2 of 4), moderate (3 of 4), and marked (4 of 4).

### Statistical analyses

Statistical analyses were performed using NCSS (NCSS, Kaysville, UT), and differences between the experimental groups were tested using one-way analysis of variance and Tukey–Kramer’s test.

## SUPPLEMENTAL MATERIAL

Supplemental material aac.00112-24-s0001.docx Tables S1 to S5; Figures S1 and S2.

ASM does not own the copyrights to Supplemental Material that may be linked to, or accessed through, an article. The authors have granted ASM a non-exclusive, world-wide license to publish the Supplemental Material files. Please contact the corresponding author directly for reuse.
