# Supplementary material for: Rapid in vitro activity of telavancin against Bacillus anthracis and in vivo protection against inhalation anthrax infection in the rabbit model
Source: Antimicrob Agents Chemother. 2024 Jun 18;68(7):e00112-24. doi: 10.1128/aac.00112-24 (PMC11232409; doi:10.1128/aac.00112-24)
Supplement: Supplemental material — Tables S1 to S5; Figures S1 and S2. [file aac.00112-24-s0001.docx]

**Supplemental Materials**

**S1 Table. Group survival percentages**

**S2 Table. Group temperature data**

**S3 Table. Bacteremia data at 24 hours post-infection**

**S4 Table. Lung bacterial load data**

**S5 Table. Anti-PA antibody data**

**S1 Fig. Temperature response during infection and antibiotic treatment.**

**S2 Fig. Histopathological lesions in liver, heart, kidneys, and brain of rabbits infected with inhalation anthrax and treated with telavancin and levofloxacin.**

**S1 Table. Group survival percentages**

|  | **Percent Survival (%)** | | |
| --- | --- | --- | --- |
| **Days post-infection** | **Telavancin** | **Levofloxacin** | **Saline** |
| 0 | 100 | 100 | 100 |
| 0.5 | 100 | 100 | 100 |
| 1 | 100 | 100 | 100 |
| 1.5 | 100 | 100 | 100 |
| 2 | 100 | 100 | 83 |
| 2.5 | 100 | 100 | 33 |
| 3 | 100 | 100 | 17 |
| 3.5 | 100 | 100 | 17 |
| 4 | 100 | 100 | 17 |
| 4.5 | 100 | 100 | 0 |
| 5 | 100 | 100 | 0 |
| 5.5 | 100 | 100 | 0 |
| 6 | 100 | 100 | 0 |
| 6.5 | 100 | 100 | 0 |
| 7 | 100 | 100 | 0 |
| 7.5 | 100 | 100 | 0 |
| 8 | 100 | 100 | 0 |
| 8.5 | 100 | 100 | 0 |
| 9 | 100 | 100 | 0 |
| 9.5 | 100 | 100 | 0 |
| 10 | 100 | 100 | 0 |
| 10.5 | 100 | 100 | 0 |
| 11 | 100 | 100 | 0 |
| 11.5 | 100 | 100 | 0 |
| 12 | 100 | 100 | 0 |
| 12.5 | 100 | 100 | 0 |
| 13 | 100 | 100 | 0 |
| 13.5 | 100 | 100 | 0 |
| 14 | 100 | 100 | 0 |

**S2 Table. Group temperature data**

|  | **Average Temperature (C˚)** | | |
| --- | --- | --- | --- |
| **Days from infection** | **Telavancin** | **Levofloxacin** | **Saline** |
| -7.00 | 39.1 | 39.2 | 39.3 |
| -6.92 | 39.2 | 39.1 | 39.3 |
| -6.83 | 39.2 | 39.2 | 39.2 |
| -6.75 | 39.3 | 39.2 | 39.3 |
| -6.67 | 39.3 | 39.3 | 39.3 |
| -6.58 | 39.2 | 39.1 | 39.2 |
| -6.50 | 39.1 | 39.1 | 39.1 |
| -6.42 | 39.1 | 39.0 | 39.1 |
| -6.33 | 39.2 | 39.1 | 39.2 |
| -6.25 | 39.2 | 39.1 | 39.3 |
| -6.17 | 39.2 | 39.1 | 39.2 |
| -6.08 | 39.1 | 39.1 | 39.2 |
| -6.00 | 39.1 | 39.1 | 39.1 |
| -5.92 | 39.0 | 39.0 | 39.1 |
| -5.83 | 39.0 | 39.0 | 39.2 |
| -5.75 | 39.0 | 39.1 | 39.1 |
| -5.67 | 39.2 | 39.1 | 39.1 |
| -5.58 | 39.2 | 39.2 | 39.2 |
| -5.50 | 39.2 | 39.2 | 39.3 |
| -5.42 | 39.3 | 39.2 | 39.3 |
| -5.33 | 39.3 | 39.2 | 39.3 |
| -5.25 | 39.2 | 39.3 | 39.3 |
| -5.17 | 39.3 | 39.3 | 39.3 |
| -5.08 | 39.2 | 39.2 | 39.3 |
| -5.00 | 39.2 | 39.1 | 39.3 |
| -4.92 | 39.0 | 39.0 | 39.2 |
| -4.83 | 39.0 | 39.0 | 39.2 |
| -4.75 | 39.1 | 39.1 | 39.1 |
| -4.67 | 39.2 | 39.1 | 39.2 |
| -4.58 | 39.2 | 39.1 | 39.3 |
| -4.50 | 39.2 | 39.2 | 39.3 |
| -4.42 | 39.2 | 39.2 | 39.3 |
| -4.33 | 39.2 | 39.2 | 39.3 |
| -4.25 | 39.2 | 39.3 | 39.3 |
| -4.17 | 39.2 | 39.2 | 39.2 |
| -4.08 | 39.2 | 39.2 | 39.4 |
| -4.00 | 39.2 | 39.2 | 39.2 |
| -3.92 | 39.1 | 39.1 | 39.2 |
| -3.83 | 39.0 | 39.0 | 39.3 |
| -3.75 | 39.1 | 39.1 | 39.2 |
| -3.67 | 39.2 | 39.1 | 39.2 |
| -3.58 | 39.1 | 39.1 | 39.3 |
| -3.50 | 39.1 | 39.1 | 39.3 |
| -3.42 | 39.2 | 39.1 | 39.3 |
| -3.33 | 39.1 | 39.2 | 39.3 |
| -3.25 | 39.2 | 39.1 | 39.4 |
| -3.17 | 39.2 | 39.1 | 39.2 |
| -3.08 | 39.1 | 39.0 | 39.3 |
| -3.00 | 39.0 | 39.0 | 39.2 |
| -2.92 | 38.9 | 38.9 | 39.2 |
| -2.83 | 38.9 | 38.9 | 39.1 |
| -2.75 | 38.9 | 39.0 | 39.0 |
| -2.67 | 39.0 | 39.0 | 39.2 |
| -2.58 | 39.1 | 39.0 | 39.2 |
| -2.50 | 39.1 | 39.0 | 39.3 |
| -2.42 | 39.1 | 39.1 | 39.3 |
| -2.33 | 39.2 | 39.1 | 39.4 |
| -2.25 | 39.2 | 39.2 | 39.4 |
| -2.17 | 39.2 | 39.2 | 39.3 |
| -2.08 | 39.1 | 39.1 | 39.4 |
| -2.00 | 39.0 | 39.0 | 39.2 |
| -1.92 | 38.9 | 38.9 | 39.2 |
| -1.83 | 38.9 | 38.9 | 39.1 |
| -1.75 | 39.0 | 39.0 | 39.1 |
| -1.67 | 39.1 | 39.1 | 39.1 |
| -1.58 | 39.2 | 39.1 | 39.3 |
| -1.50 | 39.2 | 39.1 | 39.2 |
| -1.42 | 39.2 | 39.1 | 39.3 |
| -1.33 | 39.2 | 39.2 | 39.3 |
| -1.25 | 39.2 | 39.1 | 39.3 |
| -1.17 | 39.3 | 39.2 | 39.4 |
| -1.08 | 39.2 | 39.1 | 39.3 |
| -1.00 | 39.1 | 39.1 | 39.3 |
| -0.92 | 39.1 | 39.0 | 39.2 |
| -0.83 | 39.0 | 39.0 | 39.1 |
| -0.75 | 39.1 | 39.1 | 39.2 |
| -0.67 | 39.2 | 39.1 | 39.2 |
| -0.58 | 39.2 | 39.2 | 39.3 |
| -0.50 | 39.2 | 39.1 | 39.3 |
| -0.42 | 39.2 | 39.1 | 39.4 |
| -0.33 | 39.2 | 39.2 | 39.3 |
| -0.25 | 39.2 | 39.2 | 39.3 |
| -0.17 | 39.3 | 39.3 | 39.3 |
| -0.08 | 39.3 | 39.2 | 39.4 |
| 0.00 | 39.3 | 39.1 | 39.3 |
| 0.08 | 38.4 | 38.2 | 38.3 |
| 0.17 | 38.9 | 38.9 | 39.0 |
| 0.25 | 39.0 | 39.0 | 39.1 |
| 0.33 | 39.1 | 39.1 | 39.2 |
| 0.42 | 39.1 | 39.1 | 39.4 |
| 0.50 | 39.2 | 39.2 | 39.4 |
| 0.58 | 39.2 | 39.3 | 39.5 |
| 0.67 | 39.2 | 39.3 | 39.5 |
| 0.75 | 39.3 | 39.3 | 39.5 |
| 0.83 | 39.3 | 39.2 | 39.5 |
| 0.92 | 39.4 | 39.3 | 39.5 |
| 1.00 | 39.6 | 39.3 | 39.6 |
| 1.08 | 40.1 | 39.7 | 39.8 |
| 1.17 | 40.4 | 40.3 | 40.3 |
| 1.25 | 40.7 | 40.7 | 41.0 |
| 1.33 | 40.8 | 40.6 | 41.4 |
| 1.42 | 40.7 | 40.4 | 41.4 |
| 1.50 | 40.4 | 40.2 | 41.0 |
| 1.58 | 40.0 | 40.0 | 40.5 |
| 1.67 | 39.7 | 39.9 | 40.5 |
| 1.75 | 39.6 | 39.7 | 40.7 |
| 1.83 | 39.6 | 39.5 | 40.9 |
| 1.92 | 39.5 | 39.3 | 40.9 |
| 2.00 | 39.3 | 39.2 | 40.5 |
| 2.08 | 39.2 | 39.1 | 40.3 |
| 2.17 | 38.9 | 39.0 | 40.4 |
| 2.25 | 38.8 | 39.0 | 40.2 |
| 2.33 | 38.8 | 39.0 | 40.6 |
| 2.42 | 38.8 | 39.1 | 40.6 |
| 2.50 | 38.8 | 39.1 | 40.4 |
| 2.58 | 38.7 | 39.1 | 40.0 |
| 2.67 | 38.7 | 39.1 | 39.8 |
| 2.75 | 38.8 | 39.1 | 41.7 |
| 2.83 | 39.0 | 39.1 | 41.7 |
| 2.92 | 39.1 | 39.2 | 41.3 |
| 3.00 | 39.1 | 39.1 | 41.3 |
| 3.08 | 39.1 | 39.1 | 41.2 |
| 3.17 | 38.9 | 39.1 | 40.1 |
| 3.25 | 38.9 | 39.1 | 38.5 |
| 3.33 | 39.0 | 39.1 | 39.4 |
| 3.42 | 39.2 | 39.2 | 40.5 |
| 3.50 | 39.1 | 39.2 | 40.2 |
| 3.58 | 39.0 | 39.2 | 39.5 |
| 3.67 | 39.0 | 39.2 | 39.3 |
| 3.75 | 39.1 | 39.2 | 38.9 |
| 3.83 | 39.2 | 39.2 | 38.6 |
| 3.92 | 39.1 | 39.1 | 38.4 |
| 4.00 | 39.1 | 39.1 | 37.9 |
| 4.08 | 39.0 | 39.1 | 37.3 |
| 4.17 | 39.0 | 39.1 | 36.8 |
| 4.25 | 39.0 | 39.1 | --- |
| 4.33 | 39.1 | 39.1 | --- |
| 4.42 | 39.2 | 39.1 | --- |
| 4.50 | 39.3 | 39.2 | --- |
| 4.58 | 39.3 | 39.2 | --- |
| 4.67 | 39.3 | 39.2 | --- |
| 4.75 | 39.3 | 39.2 | --- |
| 4.83 | 39.3 | 39.2 | --- |
| 4.92 | 39.3 | 39.2 | --- |
| 5.00 | 39.4 | 39.1 | --- |
| 5.08 | 39.3 | 39.0 | --- |
| 5.17 | 39.2 | 39.0 | --- |
| 5.25 | 39.2 | 39.1 | --- |
| 5.33 | 39.2 | 39.1 | --- |
| 5.42 | 39.2 | 39.2 | --- |
| 5.50 | 39.2 | 39.2 | --- |
| 5.58 | 39.1 | 39.3 | --- |
| 5.67 | 39.1 | 39.2 | --- |
| 5.75 | 39.0 | 39.2 | --- |
| 5.83 | 39.1 | 39.2 | --- |
| 5.92 | 39.2 | 39.2 | --- |
| 6.00 | 39.2 | 39.2 | --- |
| 6.08 | 39.1 | 39.1 | --- |
| 6.17 | 39.1 | 39.1 | --- |
| 6.25 | 39.0 | 39.1 | --- |
| 6.33 | 39.1 | 39.2 | --- |
| 6.42 | 39.2 | 39.3 | --- |
| 6.50 | 39.2 | 39.4 | --- |
| 6.58 | 39.2 | 39.5 | --- |
| 6.67 | 39.3 | 39.4 | --- |
| 6.75 | 39.2 | 39.4 | --- |
| 6.83 | 39.2 | 39.4 | --- |
| 6.92 | 39.2 | 39.5 | --- |
| 7.00 | 39.2 | 39.7 | --- |
| 7.08 | 39.2 | 39.6 | --- |
| 7.17 | 39.2 | 39.6 | --- |
| 7.25 | 39.2 | 39.5 | --- |
| 7.33 | 39.3 | 39.4 | --- |
| 7.42 | 39.3 | 39.5 | --- |
| 7.50 | 39.3 | 39.5 | --- |
| 7.58 | 39.5 | 39.4 | --- |
| 7.67 | 39.6 | 39.4 | --- |
| 7.75 | 39.6 | 39.3 | --- |
| 7.83 | 39.6 | 39.3 | --- |
| 7.92 | 39.6 | 39.3 | --- |
| 8.00 | 39.5 | 39.4 | --- |
| 8.08 | 39.4 | 39.3 | --- |
| 8.17 | 39.2 | 39.3 | --- |
| 8.25 | 39.2 | 39.3 | --- |
| 8.33 | 39.3 | 39.3 | --- |
| 8.42 | 39.3 | 39.4 | --- |
| 8.50 | 39.3 | 39.4 | --- |
| 8.58 | 39.3 | 39.4 | --- |
| 8.67 | 39.3 | 39.6 | --- |
| 8.75 | 39.3 | 39.6 | --- |
| 8.83 | 39.3 | 39.6 | --- |
| 8.92 | 39.3 | 39.6 | --- |
| 9.00 | 39.2 | 39.6 | --- |
| 9.08 | 39.2 | 39.5 | --- |
| 9.17 | 39.2 | 39.5 | --- |
| 9.25 | 39.3 | 39.4 | --- |
| 9.33 | 39.5 | 39.5 | --- |
| 9.42 | 39.5 | 39.5 | --- |
| 9.50 | 39.5 | 39.4 | --- |
| 9.58 | 39.5 | 39.5 | --- |
| 9.67 | 39.5 | 39.4 | --- |
| 9.75 | 39.5 | 39.4 | --- |
| 9.83 | 39.4 | 39.4 | --- |
| 9.92 | 39.4 | 39.4 | --- |
| 10.00 | 39.2 | 39.5 | --- |
| 10.08 | 39.3 | 39.4 | --- |
| 10.17 | 39.3 | 39.5 | --- |
| 10.25 | 39.3 | 39.4 | --- |
| 10.33 | 39.3 | 39.3 | --- |
| 10.42 | 39.3 | 39.2 | --- |
| 10.50 | 39.3 | 39.2 | --- |
| 10.58 | 39.3 | 39.3 | --- |
| 10.67 | 39.3 | 39.2 | --- |
| 10.75 | 39.3 | 39.2 | --- |
| 10.83 | 39.3 | 39.2 | --- |
| 10.92 | 39.3 | 39.2 | --- |
| 11.00 | 39.2 | 39.1 | --- |
| 11.08 | 39.1 | 39.1 | --- |
| 11.17 | 39.1 | 39.1 | --- |
| 11.25 | 39.2 | 39.1 | --- |
| 11.33 | 39.2 | 39.1 | --- |
| 11.42 | 39.3 | 39.1 | --- |
| 11.50 | 39.3 | 39.2 | --- |
| 11.58 | 39.3 | 39.2 | --- |
| 11.67 | 39.3 | 39.2 | --- |
| 11.75 | 39.3 | 39.2 | --- |
| 11.83 | 39.3 | 39.2 | --- |
| 11.92 | 39.3 | 39.2 | --- |
| 12.00 | 39.2 | 39.1 | --- |
| 12.08 | 39.2 | 39.1 | --- |
| 12.17 | 39.2 | 39.0 | --- |
| 12.25 | 39.2 | 39.1 | --- |
| 12.33 | 39.3 | 39.2 | --- |
| 12.42 | 39.4 | 39.3 | --- |
| 12.50 | 39.4 | 39.2 | --- |
| 12.58 | 39.3 | 39.3 | --- |
| 12.67 | 39.3 | 39.2 | --- |
| 12.75 | 39.3 | 39.2 | --- |
| 12.83 | 39.3 | 39.2 | --- |
| 12.92 | 39.3 | 39.2 | --- |
| 13.00 | 39.2 | 39.2 | --- |
| 13.08 | 39.2 | 39.1 | --- |
| 13.17 | 39.2 | 39.1 | --- |
| 13.25 | 39.2 | 39.1 | --- |
| 13.33 | 39.3 | 39.2 | --- |
| 13.42 | 39.3 | 39.2 | --- |
| 13.50 | 39.3 | 39.2 | --- |
| 13.58 | 39.3 | 39.2 | --- |
| 13.67 | 39.2 | 39.3 | --- |
| 13.75 | 39.3 | 39.2 | --- |
| 13.83 | 39.3 | 39.2 | --- |
| 13.92 | 39.3 | 39.2 | --- |
| 14.00 | 39.0 | 39.1 | --- |

**S3 Table. Bacteremia data at 24 hours post-infection**

| **Group** | **Animal ID#** | **Bacterial count (cfu/ml)** | **Log-transformed count (cfu/ml)** | **Average log count (cfu/ml)** | **Standard error (cfu/ml)** |
| --- | --- | --- | --- | --- | --- |
| Telavancin | 8629 | 0 | 0 | 0.37 | 0.25 |
|  | 8630 | 0 | 0 |  |  |
|  | 8633 | 0 | 0 |  |  |
|  | 8637 | 0 | 0 |  |  |
|  | 8639 | 0 | 0 |  |  |
|  | 8641 | 0 | 0 |  |  |
|  | 8643 | 0 | 0 |  |  |
|  | 8646 | 3.90E+02 | 2.59 |  |  |
|  | 8647 | 0 | 0 |  |  |
|  | 8651 | 0 | 0 |  |  |
|  | 8653 | 7.00E+01 | 1.85 |  |  |
|  | 8656 | 0 | 0 |  |  |
| Levofloxacin | 8628 | 1.00E+01 | 1.00 | 1.14 | 0.33 |
|  | 8632 | 7.00E+01 | 1.85 |  |  |
|  | 8634 | 0 | 0 |  |  |
|  | 8635 | 1.00E+01 | 1.00 |  |  |
|  | 8638 | 0 | 0 |  |  |
|  | 8640 | 0 | 0 |  |  |
|  | 8642 | 2.00E+01 | 1.30 |  |  |
|  | 8644 | 0 | 0 |  |  |
|  | 8649 | 4.42E+03 | 3.65 |  |  |
|  | 8652 | 3.60E+02 | 2.56 |  |  |
|  | 8654 | 2.00E+01 | 1.30 |  |  |
|  | 8657 | 1.00E+01 | 1.00 |  |  |
| Saline | 8631 | 1.90E+05 | 5.28 | 2.59 | 0.90 |
|  | 8636 | 0 | 0 |  |  |
|  | 8645 | 6.71E+03 | 3.83 |  |  |
|  | 8648 | 2.50E+02 | 2.40 |  |  |
|  | 8650 | 1.16E+04 | 4.06 |  |  |
|  | 8655 | 0 | 0 |  |  |

**S4 Table. Lung bacterial load data**

| **Group** | **Animal ID#** | **Bacterial count (cfu/g)** | **Log-transformed count (cfu/g)** | **Average log count (cfu/g)** | **Standard error (cfu/g)** |
| --- | --- | --- | --- | --- | --- |
| Telavancin | 8629 | 1.83E+03 | 3.26 | 3.19 | 0.09 |
|  | 8630 | 5.03E+02 | 2.70 |  |  |
|  | 8633 | 7.71E+02 | 2.89 |  |  |
|  | 8637 | 8.03E+02 | 2.90 |  |  |
|  | 8639 | 1.60E+03 | 3.20 |  |  |
|  | 8641 | 2.25E+03 | 3.35 |  |  |
|  | 8643 | 1.07E+03 | 3.03 |  |  |
|  | 8646 | 1.18E+03 | 3.07 |  |  |
|  | 8647 | 4.52E+03 | 3.66 |  |  |
|  | 8651 | 1.74E+03 | 3.24 |  |  |
|  | 8653 | 5.08E+03 | 3.71 |  |  |
|  | 8656 | 1.78E+03 | 3.25 |  |  |
| Levofloxacin | 8628 | 2.88E+04 | 4.46 | 3.60 | 0.14 |
|  | 8632 | 3.43E+03 | 3.54 |  |  |
|  | 8634 | 2.69E+03 | 3.43 |  |  |
|  | 8635 | 2.77E+03 | 3.44 |  |  |
|  | 8638 | 3.50E+03 | 3.54 |  |  |
|  | 8640 | 8.72E+02 | 2.94 |  |  |
|  | 8642 | 9.98E+03 | 4.00 |  |  |
|  | 8644 | 1.24E+04 | 4.09 |  |  |
|  | 8649 | 4.96E+03 | 3.70 |  |  |
|  | 8652 | 1.76E+03 | 3.25 |  |  |
|  | 8654 | 5.37E+02 | 2.73 |  |  |
|  | 8657 | 1.15E+04 | 4.06 |  |  |
| Saline | 8631 | 1.51E+06 | 6.18 | 6.28 | 0.21 |
|  | 8636 | 5.68E+06 | 6.75 |  |  |
|  | 8645 | 3.49E+06 | 6.54 |  |  |
|  | 8648 | 2.39E+05 | 5.38 |  |  |
|  | 8650 | 1.21E+06 | 6.08 |  |  |
|  | 8655 | 5.26E+06 | 6.72 |  |  |

**S5 Table. Anti-PA antibody data**

| **Group** | **Days post-infection** | **Animal ID#** | **Fold increase in signal** | **Average fold increase in signal** | **Standard error** |
| --- | --- | --- | --- | --- | --- |
| Telavancin | 3 days | 8629 | 1.26 | 1.24 | 0.21 |
|  |  | 8630 | 1.53 |  |  |
|  |  | 8633 | 2.92 |  |  |
|  |  | 8637 | 0.79 |  |  |
|  |  | 8639 | 2.29 |  |  |
|  |  | 8641 | 0.54 |  |  |
|  |  | 8643 | 0.90 |  |  |
|  |  | 8646 | 0.97 |  |  |
|  |  | 8647 | 0.44 |  |  |
|  |  | 8651 | 1.37 |  |  |
|  |  | 8653 | 1.17 |  |  |
|  |  | 8656 | 0.73 |  |  |
|  | 7 days | 8629 | 321.97 | 73.04 | 26.17 |
|  |  | 8630 | 11.43 |  |  |
|  |  | 8633 | 58.44 |  |  |
|  |  | 8637 | 3.31 |  |  |
|  |  | 8639 | 86.25 |  |  |
|  |  | 8641 | 11.65 |  |  |
|  |  | 8643 | 143.63 |  |  |
|  |  | 8646 | 54.04 |  |  |
|  |  | 8647 | 8.54 |  |  |
|  |  | 8651 | 118.96 |  |  |
|  |  | 8653 | 21.29 |  |  |
|  |  | 8656 | 36.95 |  |  |
|  | 10 days | 8629 | 497.78 | 358.28 | 67.58 |
|  |  | 8630 | 197.45 |  |  |
|  |  | 8633 | 388.88 |  |  |
|  |  | 8637 | 4.77 |  |  |
|  |  | 8639 | 533.56 |  |  |
|  |  | 8641 | 323.65 |  |  |
|  |  | 8643 | 662.89 |  |  |
|  |  | 8646 | 392.94 |  |  |
|  |  | 8647 | 64.15 |  |  |
|  |  | 8651 | 785.97 |  |  |
|  |  | 8653 | 219.01 |  |  |
|  |  | 8656 | 228.37 |  |  |
|  | 14 days | 8629 | 466.74 | 449.94 | 79.42 |
|  |  | 8630 | 221.07 |  |  |
|  |  | 8633 | 267.35 |  |  |
|  |  | 8637 | 18.28 |  |  |
|  |  | 8639 | 440.92 |  |  |
|  |  | 8641 | 647.13 |  |  |
|  |  | 8643 | 686.58 |  |  |
|  |  | 8646 | 426.59 |  |  |
|  |  | 8647 | 103.07 |  |  |
|  |  | 8651 | 984.65 |  |  |
|  |  | 8653 | 691.65 |  |  |
|  |  | 8656 | 445.32 |  |  |
| Levofloxacin | 3 days | 8628 | 1.32 | 1.10 | 0.11 |
|  |  | 8632 | 1.47 |  |  |
|  |  | 8634 | 1.30 |  |  |
|  |  | 8635 | 0.70 |  |  |
|  |  | 8638 | 1.52 |  |  |
|  |  | 8640 | 1.52 |  |  |
|  |  | 8642 | 0.71 |  |  |
|  |  | 8644 | 0.71 |  |  |
|  |  | 8649 | 0.88 |  |  |
|  |  | 8652 | 0.83 |  |  |
|  |  | 8654 | 0.70 |  |  |
|  |  | 8657 | 1.56 |  |  |
|  | 7 days | 8628 | 50.86 | 60.30 | 20.22 |
|  |  | 8632 | 81.27 |  |  |
|  |  | 8634 | 11.93 |  |  |
|  |  | 8635 | 1.73 |  |  |
|  |  | 8638 | 10.22 |  |  |
|  |  | 8640 | 7.75 |  |  |
|  |  | 8642 | 2.07 |  |  |
|  |  | 8644 | 1.96 |  |  |
|  |  | 8649 | 223.25 |  |  |
|  |  | 8652 | 98.63 |  |  |
|  |  | 8654 | 92.78 |  |  |
|  |  | 8657 | 141.18 |  |  |
|  | 10 days | 8628 | 412.90 | 474.83 | 118.21 |
|  |  | 8632 | 355.00 |  |  |
|  |  | 8634 | 738.56 |  |  |
|  |  | 8635 | 13.72 |  |  |
|  |  | 8638 | 165.87 |  |  |
|  |  | 8640 | 418.21 |  |  |
|  |  | 8642 | 19.19 |  |  |
|  |  | 8644 | 147.69 |  |  |
|  |  | 8649 | 556.43 |  |  |
|  |  | 8652 | 426.13 |  |  |
|  |  | 8654 | 1171.57 |  |  |
|  |  | 8657 | 1272.64 |  |  |
|  | 14 days | 8628 | 411.40 | 662.53 | 114.85 |
|  |  | 8632 | 347.14 |  |  |
|  |  | 8634 | 783.60 |  |  |
|  |  | 8635 | 569.91 |  |  |
|  |  | 8638 | 859.63 |  |  |
|  |  | 8640 | 1190.93 |  |  |
|  |  | 8642 | 67.58 |  |  |
|  |  | 8644 | 178.49 |  |  |
|  |  | 8649 | 541.00 |  |  |
|  |  | 8652 | 559.08 |  |  |
|  |  | 8654 | 1207.67 |  |  |
|  |  | 8657 | 1233.94 |  |  |
| Saline | 3 days | 8631 | 0 | 0.64 | 0.37 |
|  |  | 8636 | 1.84 |  |  |
|  |  | 8645 | 1.18 |  |  |
|  |  | 8648 | 0.07 |  |  |
|  |  | 8650 | 0.07 |  |  |
|  |  | 8655 | 0.02 |  |  |
|  | 7 days | 8631 | --- | --- | --- |
|  |  | 8636 | --- |  |  |
|  |  | 8645 | --- |  |  |
|  |  | 8648 | --- |  |  |
|  |  | 8650 | --- |  |  |
|  |  | 8655 | --- |  |  |
|  | 10 days | 8631 | --- | --- | --- |
|  |  | 8636 | --- |  |  |
|  |  | 8645 | --- |  |  |
|  |  | 8648 | --- |  |  |
|  |  | 8650 | --- |  |  |
|  |  | 8655 | --- |  |  |
|  | 14 days | 8631 | --- | --- | --- |
|  |  | 8636 | --- |  |  |
|  |  | 8645 | --- |  |  |
|  |  | 8648 | --- |  |  |
|  |  | 8650 | --- |  |  |
|  |  | 8655 | --- |  |  |


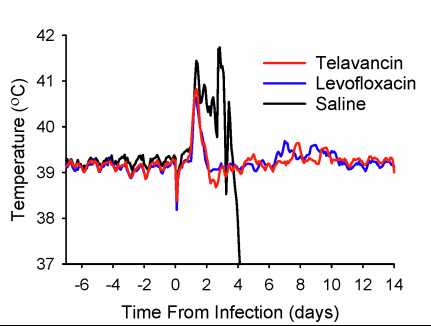


**S1 Fig. Temperature response during infection and antibiotic treatment.** New Zealand White rabbits were challenged with 225 LD_50_ *B. anthracis* Ames spores via inhalation. Telavancin treatment was initiated upon detection of PA in the animals’ sera and was administered at 30 mg/kg twice daily for 5 days. Levofloxacin, at 12.5 mg/kg, administered once daily for 5 days, and saline, administered once daily, were used as controls. Temperatures were recorded every 10 min for 7 days before infection and for the entire post-infection period using implantable data loggers. The data are presented as two-hour moving averages.


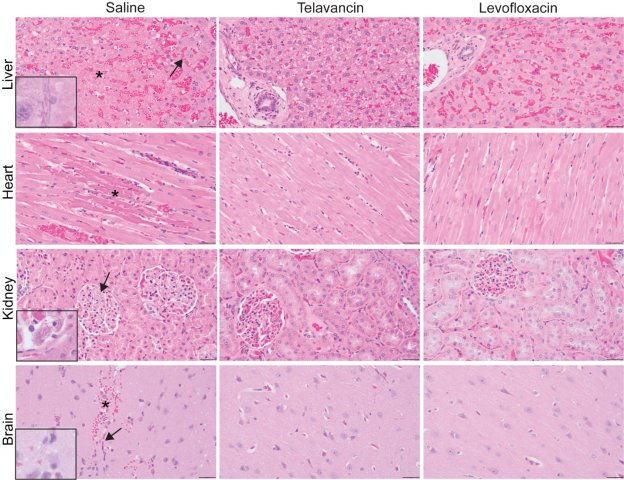


**S2 Fig. Histopathological lesions in liver, heart, kidneys, and brain of rabbits infected with inhalation anthrax and treated with telavancin and levofloxacin.** New Zealand White rabbits were inoculated with anthrax via the inhalation route. The liver, heart, kidney, and brain were collected for routine histopathology. Significant lesions were only detected in the animals dosed with saline. Liver: The liver has random areas of hepatocellular necrosis (asterisk) with intrasinusoidal bacteria (arrow). Inset, higher magnification of anthrax bacilli within the sinusoid. Heart: The cardiomyocytes are focally necrotic (asterisk). Kidney: The glomerular capillaries contain intravascular bacteria (arrow). Inset, higher magnification of anthrax bacilli within the capillary loops. Brain. Within the gray matter of the cortex are areas of hemorrhage (*) and intravascular bacteria (arrow). Inset, higher magnification of anthrax bacilli. Hematoxylin and eosin. Scale bar = 20 µm.
